# NTS Neural Subtypes and Satiety Pathways: taVNS as a Novel Neuromodulatory Intervention

**DOI:** 10.3390/brainsci16070755

**Published:** 2026-07-17

**Authors:** Hanyu Deng, Chen Xin, Haohan Zhu, Xinjiang Zhang, Lan Sun, Yanfeng Zheng, Peijing Rong

**Affiliations:** 1 Institute of Acupuncture and Moxibustion, China Academy of Chinese Medical Sciences, Beijing 100700, China; 2 Institute of Basic Research in Clinical Medicine, China Academy of Chinese Medical Sciences, Beijing 100700, China

**Keywords:** nucleus of the solitary tract, transcutaneous auricular vagus nerve stimulation, obesity, feeding homeostasis, neuronal subpopulations, satiety

## Abstract

**Highlights:**

**What are the main findings?**

- This narrative review provides a comprehensive delineation of the nucleus of the solitary tract (NTS) as the primary central relay for peripheral satiety signals, and clarifies the distinct roles of its heterogeneous neuronal subpopulations in feeding homeostasis.
- It summarizes preclinical and clinical evidence that transcutaneous auricular vagus nerve stimulation (taVNS) exerts anti-obesity effects by restoring impaired NTS-mediated satiety processing, filling an existing gap in the narrative literature.

**What are the implications of the main findings?**

- This work establishes an integrated conceptual framework linking NTS neurocircuit dysfunction to obesity pathogenesis, providing clear directions for future mechanistic studies on feeding regulation.
- It highlights the translational value of taVNS as a non-invasive neuromodulation strategy targeting the NTS and proposes key priorities to accelerate its clinical application in obesity management.

**Abstract:**

The global obesity epidemic has become one of the most severe public health challenges of the 21st century, and existing treatment strategies have limitations such as poor long-term adherence, significant side effects, or high invasiveness. Dysregulation of feeding behavior is the core driver of obesity, and its precise regulation depends on the complete transmission of gut–brain axis signals. The nucleus of the solitary tract (NTS), as the first central relay station for visceral afferent signals, is a key hub for the regulation of feeding homeostasis. This article reviews the peripheral signal transduction mechanisms of hunger and satiety, the core afferent pathway of the vagus nerve, and the functional heterogeneity of neuron subpopulations within the NTS, focusing on the anatomical basis, neuroimaging evidence, and preclinical and clinical research progress of transcutaneous auricular vagus nerve stimulation (taVNS) targeting the NTS to exert anti-obesity effects. Accumulating evidence suggests that taVNS may modulate the activity of satiety-promoting neurons and attenuate aberrant hyperactivity of TH^+^ catecholaminergic neurons in obesity via the auricular branch of the vagus nerve–NTS pathway, which may in turn bidirectionally suppress homeostatic and hedonic feeding, reduce energy intake, and ameliorate metabolic dysregulation. Current research still has key gaps such as unstandardized stimulation parameters, lack of large-sample long-term randomized controlled trials, and unclear interaction mechanisms between the NTS and higher brain regions. This article provides a theoretical basis for in-depth understanding of the feeding regulatory network of the NTS and the translational application of taVNS in obesity management.

## 1. Introduction

Dysregulation of feeding behavior is the core driver of obesity, and its regulation depends on the precise transmission of gut–brain axis signals. The nucleus of the solitary tract (NTS), as the first relay station for visceral afferent signals entering the central nervous system, is a key hub for integrating peripheral satiety signals and coordinating homeostatic and hedonic feeding [1]. Transcutaneous auricular vagus nerve stimulation (taVNS) specifically activates the NTS by stimulating the auricular branch of the vagus nerve on the body surface, showing promising anti-obesity application prospects, but many key issues regarding its mechanism of action and clinical translation remain to be clarified. This article reviews the peripheral signal pathways of feeding regulation, the afferent mechanism of the vagus nerve, and the functional heterogeneity of NTS neuron subpopulations, focusing on the anti-obesity evidence and research gaps of taVNS targeting the NTS, to provide a theoretical basis for the clinical translation of this technology.

## 2. Literature Retrieval Strategy

This narrative review synthesizes evidence from multiple sources to construct an integrated conceptual framework of NTS-mediated feeding regulation and the therapeutic potential of taVNS.

Literature searches were performed up to 7 June 2026, across six electronic databases covering both the English and Chinese literature. The English-language literature was retrieved from PubMed, Web of Science, and Scopus. The Chinese-language literature was retrieved from three major domestic academic databases: CNKI (China National Knowledge Infrastructure), Wanfang Data, and VIP Chinese Journal Database.

For English databases, searches were performed using combinations of the following keywords: “nucleus of the solitary tract”, “nucleus tractus solitarius”, “NTS”, “vagus nerve”, “taVNS”, “transcutaneous auricular vagus nerve stimulation”, “obesity”, “feeding”, “satiety”, “neuronal subpopulations”, “GLP-1”, “CCK”, and “catecholaminergic”. Corresponding Chinese terminology was used for searches in Chinese databases. Reference lists of all identified eligible articles were manually searched to capture additional relevant studies that may have been missed by database retrieval.

Study selection was conducted independently by two investigators. First, titles and abstracts were screened against predefined relevance criteria to identify potentially eligible records. Full-text articles were then retrieved and assessed for inclusion. Disagreements regarding study eligibility were resolved through in-depth discussion and consensus between the two reviewers.

As a narrative review focused on mechanistic integration, this work does not follow the full PRISMA guidelines for systematic reviews, and no formal quantitative quality assessment scoring was applied to included studies. Instead, all included studies were evaluated for methodological rigor and relevance to the core mechanistic themes of the review. The primary aim of this work is to integrate mechanistic insights across multiple levels of analysis and identify critical research gaps, rather than to provide an exhaustive quantitative synthesis of all available evidence.

## 3. Current Status of the Relationship Between the High Prevalence of Obesity and Dysregulation of Feeding Homeostasis

### 3.1. Global Burden of Obesity Disease and Limitations of Existing Treatment Strategies

The global obesity pandemic has become one of the most severe public health challenges of the 21st century. According to data from the 2025 Global Burden of Disease (GBD) study published in The Lancet, the prevalence of overweight and obesity among adults aged 25 years and older worldwide reached 45.1% in 2021, affecting approximately 2.11 billion people, of whom about 1 billion are obese [2]. Obesity is not only an independent disease but also a major risk factor for chronic non-communicable diseases such as type 2 diabetes, cardiovascular diseases, various cancers, and musculoskeletal disorders [3]. It is estimated that obesity-related diseases cause approximately 4 million deaths globally each year, accounting for 7% of total deaths [4].

Despite the growing severity of the obesity problem, existing treatment strategies still have significant limitations. Lifestyle interventions (dietary control and exercise) are the foundation of obesity treatment, but they have poor long-term adherence, and most patients experience weight rebound after intervention. In terms of pharmacotherapy, although new-generation glucagon-like peptide-1 (GLP-1) receptor agonists such as semaglutide have shown good weight loss effects, they have problems such as gastrointestinal side effects, unknown long-term safety, high cost, and weight rebound after discontinuation [5,6]. Bariatric surgery, although having a significant weight loss effect, is invasive, carries surgical risks and postoperative complications, and is only applicable to patients with severe obesity [7]. Therefore, there is an urgent need to develop safe, effective, and highly accessible novel obesity intervention methods.

### 3.2. Dysregulation of Feeding Behavior Is the Core Driver of Obesity Development

The core driving factor of the global obesity pandemic and its related metabolic complications is dysregulation of feeding behavior [8]. Normal feeding behavior is precisely regulated by the nervous system to maintain energy balance. According to different regulatory mechanisms, feeding behavior can be divided into two categories: homeostatic feeding and hedonic feeding.

Homeostatic feeding is driven by the body’s energy requirements, with the purpose of replenishing energy consumption and maintaining the stability of physiological indicators such as blood glucose and fat reserves [9]. This process mainly relies on the precise transmission of gut–brain axis signals: during food digestion, the gastrointestinal tract secretes satiety hormones such as cholecystokinin (CCK), GLP-1, and peptide YY (PYY) [10], while gastric distension stimulates vagal afferent fibers. These signals are jointly transmitted to the central nervous system, producing satiety and terminating eating. Conversely, when the body is energy-deficient, ghrelin secreted by the stomach acts on the central nervous system, producing hunger and initiating eating behavior.

Hedonic feeding is driven by the rewarding properties of food (such as delicious taste and texture) and is independent of the body’s energy requirements. This feeding behavior is mainly regulated by the midbrain dopamine reward system. The pleasant experience of food activates brain regions such as the nucleus accumbens and ventral tegmental area, producing a strong eating motivation and leading to overeating even when energy is sufficient [11]. In modern society, the widespread availability of high-energy, highly palatable foods has made hedonic feeding an important driver of obesity development [12].

The homeostatic regulation of hunger and satiety depends on the precise transmission of gut–brain axis signals. The nucleus of the solitary tract (NTS), as the first central relay station for visceral afferent signals, is located at the hub of gut–brain axis information integration. It is important to acknowledge that obesity is a multifactorial disorder resulting from complex interactions among genetic, epigenetic, metabolic, inflammatory, environmental, behavioral, and socioeconomic factors. While dysregulation of feeding behavior mediated by the gut–brain-NTS axis is a significant contributor to obesity pathogenesis, it is by no means the sole mechanism. Narrative review of the multi-level regulatory mechanisms of NTS in feeding homeostasis and obesity is of critical significance for understanding dysregulation of feeding behavior and developing novel intervention strategies. The following sections will elaborate layer by layer from peripheral signals and vagal afferent pathways to the internal microcircuits of the NTS.

## 4. Neurophysiological Basis of Hunger and Satiety Signals

The precise regulation of feeding behavior depends on continuous bidirectional information exchange between peripheral organs and the central nervous system [13]. Peripheral organs perceive gastrointestinal filling, nutrient concentration, and energy reserve status in real time through mechanoreceptors and chemoreceptors, converting this information into neural or humoral signals for transmission to the central nervous system [14]; the central nervous system then integrates and analyzes these signals to produce hunger or satiety, thereby initiating or terminating feeding behavior [15]. In this complex regulatory network, the NTS, as the first entry point for visceral sensory information into the central nervous system, plays an irreplaceable gateway role.

### 4.1. Peripheral Signals of Hunger and Satiety

Peripheral feeding regulatory signals are mainly divided into two categories: one is mechanical signals and intestinal peptide signals derived from the gastrointestinal tract, which mainly reflect the immediate state during feeding; the other is metabolic signals derived from the blood circulation, which mainly reflect the long-term energy reserve state of the body. The two types of signals work synergistically to maintain energy balance [16].

#### 4.1.1. Gastrointestinal Signals

Gastric distension is the most direct mechanical satiety signal. When food enters the stomach, the gastric wall smooth muscle is stretched, activating vagal mechanoreceptors distributed in the gastric wall [17]. These receptors transmit signals to the medial subnucleus of the NTS through vagal afferent nerves, thereby inhibiting feeding behavior [18]. Studies have shown that the feeding inhibitory effect caused by gastric distension can be completely blocked by bilateral subdiaphragmatic vagotomy, proving that the vagus nerve is the main pathway mediating gastric distension signals [19].

Intestinal peptides are bioactive substances secreted by gastrointestinal endocrine cells, released under the stimulation of nutrients, and exert satiety effects through endocrine or paracrine modes acting on vagal nerve endings or directly entering the blood circulation. CCK is the earliest discovered intestinal satiety factor, secreted by I cells in the upper small intestine under the stimulation of fat and protein [20]. CCK activates the vagus-NTS pathway by acting on CCK1 receptors on vagal afferent nerve endings, reducing the amount of food consumed per meal and shortening the eating time [21,22]. GLP-1, secreted by L cells in the ileum and colon, not only promotes insulin secretion and inhibits glucagon release, but also strongly inhibits feeding through vagus nerve-dependent and independent pathways acting on the NTS and hypothalamus [23]. PYY, also secreted by L cells, acts on Y2 receptors in the hypothalamic arcuate nucleus (ARC) in its active form PYY3-36, inhibiting the activity of AgRP neurons and thereby reducing food intake [20]. Peripherally, neurotensin modulates gastrointestinal motility, pancreatic secretion, and nutrient absorption. Centrally, neurotensin-expressing neurons are present in several brain regions involved in feeding regulation, including the NTS, hypothalamus, and midbrain. The effects of neurotensin on feeding are complex and site-dependent: while central administration in some brain regions can stimulate feeding, NTS neurotensin appears to exert inhibitory effects on food intake, potentially through modulation of vagal afferent signaling and interactions with other satiety pathways such as GLP-1 and CCK.

#### 4.1.2. Metabolic Signals

Glucose is the main energy source of the body, and there are a large number of neurons sensitive to changes in glucose concentration in the central nervous system, including glucose-excited (GE) neurons and glucose-inhibited (GI) neurons [24]. These neurons are mainly distributed in the hypothalamic ARC, ventromedial hypothalamus (VMH), and NTS, and can directly perceive fluctuations in blood glucose levels to regulate feeding behavior. When blood glucose decreases, GI neurons are activated to promote feeding; when blood glucose increases, GE neurons are activated to inhibit feeding.

Insulin is secreted by pancreatic β cells, and its circulating level is positively correlated with blood glucose concentration and body fat content. Insulin can cross the blood–brain barrier and act on insulin receptors in the hypothalamic ARC, inhibiting the activity of AgRP/NPY neurons while enhancing the activity of POMC neurons, thereby exerting effects of inhibiting feeding and increasing energy expenditure [13,25]. Leptin is a hormone secreted by white adipocytes, and its plasma concentration is proportional to body fat mass, making it a key signal reflecting the body’s long-term energy reserves [26]. Leptin acts on leptin receptors in the hypothalamic ARC, inhibiting the expression and release of AgRP/NPY neurons [27], while stimulating POMC neurons to produce α-melanocyte-stimulating hormone (α-MSH). The latter acts on MC4R receptors in the paraventricular hypothalamus (PVH), producing a strong satiety effect.

Contrary to the above satiety signals, ghrelin is the only known peripheral orexigenic hormone, mainly secreted by X/A-like cells in the gastric fundus during fasting [28]. Ghrelin acts on GHSR receptors in the hypothalamic ARC through the blood circulation, activating AgRP/NPY neurons while inhibiting POMC neurons, thereby strongly promoting feeding and increasing fat storage [29]. In addition, ghrelin can act on GHSR receptors on vagal afferent nerve endings, transmitting hunger signals to the central nervous system through the vagus-NTS pathway [30].

## 5. The Vagus Nerve: The Core Peripheral Afferent Pathway for Feeding and Satiety Signals

The vagus nerve, as the longest and most widely distributed cranial nerve in the body, constitutes the core anatomical and functional basis of the “gut-brain axis”, responsible for transmitting mechanical and chemical signals from the gastrointestinal tract to the central nervous system [31], and is a key pathway for the short-term regulation of feeding behavior [32]. In recent years, with the deepening understanding of the anatomy and function of the vagus nerve and the development of non-invasive vagus nerve stimulation technology, targeting the vagus nerve pathway has become a new direction for the treatment of obesity and related metabolic diseases. This chapter elaborates on the anatomical basis, functional evidence, and plastic changes in obesity of the vagus nerve in the regulation of feeding homeostasis.

### 5.1. Anatomical Distribution of the Vagus Nerve Related to Feeding Regulation

#### 5.1.1. Vagal Afferent Neurons (Nodose Ganglion) and Their Peripheral Terminals in the Gastrointestinal Tract

The vagus nerve consists of approximately 80% afferent fibers and 20% efferent fibers, with the proportion of efferent fibers in the subdiaphragmatic vagus nerve increasing to 30%. The cell bodies of all vagal afferent neurons innervating the gastrointestinal tract are located in the nodose ganglion below the jugular foramen. The axons emitted by these neurons form the vagus nerve trunk, which enters the abdominal cavity through the thoracic cavity and branches to innervate most of the digestive tract from the esophagus to the transverse colon [33].

There are significant differences in the density of vagal afferent innervation in different regions of the gastrointestinal tract. The gastric fundus and gastric body receive the highest density of vagal afferent innervation, which is consistent with the function of the stomach as a food storage organ and mechanoreceptor [34,35]. Although the small intestine has a lower nerve innervation density per unit area than the stomach, it receives the largest total number of vagal afferent nerve fibers due to its huge surface area. The vagal afferent nerve endings in the gastrointestinal tract are mainly divided into two categories: intramuscular arrays (IMAs) located in the muscular layer and mucosal endings located in the mucosal layer [32]. IMAs mainly act as mechanoreceptors, sensing the expansion and contraction of the gastrointestinal tract; while mucosal endings act as chemoreceptors, detecting nutrients and hormone signals in the intestinal lumen.

Vagal afferent nerve endings express a variety of receptors that can recognize various hormones and neurotransmitters secreted by the gastrointestinal tract, including CCK receptor A (CCK-AR), GLP-1 receptor (GLP-1R), PYY receptor Y2 (Y2R), leptin receptor (Ob-Rb), and growth hormone secretagogue receptor (GHSR). The expression of these receptors enables vagal afferent nerves to accurately perceive the nutritional status of the gastrointestinal tract and transmit this information to the central nervous system [32].

#### 5.1.2. Central Projection of Vagal Afferent Fibers: Specific Termination in the Medullary Nucleus of the Solitary Tract (NTS)

After entering the medulla oblongata, the vast majority of vagal afferent fibers specifically terminate in the NTS of the dorsal vagal complex (DVC) [36]. The NTS is a long strip-shaped nucleus extending along the entire length of the dorsal medulla oblongata, which can be divided into three parts: rostral, intermediate, and caudal according to its positional relationship with the fourth ventricle. Among them, the intermediate and caudal NTS mainly receive vagal afferent fiber projections from the gastrointestinal tract.

After receiving vagal afferent signals, NTS neurons transmit information through two main pathways: one is local projection to the dorsal motor nucleus of the vagus (DMV), forming a vagus–vagus reflex arc to regulate gastrointestinal motility, secretion, and absorption functions [37]; the other is ascending projection to higher brain regions, including the hypothalamic ARC, PVH, amygdala, insula, and prefrontal cortex, which are involved in the regulation of feeding behavior, emotion, and cognitive function [26].

### 5.2. Regulation of Hunger and Satiety by the Vagus Nerve

#### 5.2.1. Vagal Afferent Fibers Mediate Mechanical (Gastric Distension) and Chemical (Intestinal Peptide) Satiety Signals

Vagal afferent fibers mediate satiety signals through two modes: mechanoreceptors and chemoreceptors [38]. Mechanoreceptors are mainly located in the muscular layer of the gastric wall and can sense the degree of gastric distension. When food enters the stomach and causes gastric distension, mechanoreceptors are activated, and the generated nerve impulses are transmitted to the NTS through vagal afferent fibers, thereby inhibiting feeding behavior [39]. Early studies demonstrated that subdiaphragmatic vagotomy substantially attenuates the feeding inhibitory effect of gastric distension, indicating that vagal afferent fibers are a major pathway mediating this satiety signal [40]. Additionally, the completeness of vagal denervation varies across surgical procedures, and residual vagal fibers or non-vagal pathways may contribute to satiety signaling under certain conditions.

Chemoreceptors are mainly located in the mucosal layer of the gastrointestinal tract and can recognize nutrients in the intestinal lumen and various satiety hormones secreted by the gastrointestinal tract [41]. Among them, CCK is the most thoroughly studied satiety hormone, and its anorectic effect is completely dependent on vagal afferent fibers. The classic experiment by Smith et al. demonstrated that abdominal vagotomy could completely block the feeding inhibition caused by peripheral administration of CCK in rats [42]. Further studies found that CCK increases the firing frequency of vagal afferent nerves by activating CCK-AR receptors on vagal afferent nerve endings, thereby transmitting satiety signals to the NTS [43].

In addition to CCK, various intestinal peptides and adipokines such as GLP-1, PYY3-36, and leptin also exert satiety effects through vagal afferent fibers. GLP-1 is secreted by intestinal L cells, and its anorectic effect is significantly attenuated in vagotomized rats and humans. PYY3-36, secreted by L cells in the ileum and colon, can activate Y2 receptors on vagal afferent nerves to inhibit feeding behavior [44]. Leptin is mainly secreted by adipose tissue, but a small amount is also secreted by the gastric mucosa. Gastric-derived leptin can act on leptin receptors on vagal afferent nerve endings, increasing the sensitivity of vagal afferent nerves to gastric distension and CCK.

Contrary to satiety signals, the hunger hormone ghrelin is secreted by X/A cells in the stomach and can activate GHSR receptors on vagal afferent nerves, inhibiting the firing frequency of vagal afferent nerves and thereby promoting feeding behavior [45]. Studies have shown that vagotomy can completely block the increase in food intake caused by ghrelin in rats, proving that vagal afferent fibers are a key pathway mediating ghrelin hunger signals [46].

#### 5.2.2. Vagotomy Disrupts Satiety Signal Transmission and Induces Binge Eating and Obesity

Vagotomy can significantly disrupt the transmission of satiety signals, leading to binge eating and obesity in animals. Early studies found that rats with abdominal vagotomy had significantly increased food intake and faster weight gain than sham-operated rats [47]. Vagotomized rats completely lost or significantly attenuated their responses to satiety signals such as CCK, GLP-1, and gastric distension, while their responses to ghrelin hunger signals were also inhibited.

There are differences in the effects of vagotomy at different sites on feeding behavior. Subdiaphragmatic vagal trunk transection has the most significant effect on feeding and body weight, while selective transection of the gastric or hepatic branch has a relatively small effect [48]. This indicates that vagal afferent signals from different parts of the gastrointestinal tract have synergistic effects in feeding regulation.

In addition, vagotomy can also affect gastrointestinal motility and secretion functions, leading to delayed gastric emptying, decreased gastric acid secretion, and impaired nutrient absorption. These factors may also affect body weight, but studies have shown that vagotomy still leads to increased food intake and weight gain even when gastrointestinal dysfunction is controlled.

However, these findings should be interpreted with several methodological caveats. The extent of vagal denervation varies considerably across studies, ranging from selective gastric branch transection to total subdiaphragmatic vagotomy, which directly affects the magnitude of observed effects. Collectively, while animal evidence strongly supports the vagus nerve as a critical pathway for satiety signaling, the complexity of vagotomy effects cautions against oversimplified causal inferences.

#### 5.2.3. Impaired Vagus Nerve Integrity in Obese Patients Is Associated with Binge Eating Behavior

Clinical studies have also confirmed the important role of the vagus nerve in human feeding regulation [49]. Early studies found that patients who underwent vagotomy for ulcer disease often experienced weight gain after surgery [50], which is consistent with the results of animal experiments. In recent years, with the development of non-invasive vagus nerve function detection technology, more and more studies have shown that obese patients have abnormal vagus nerve function.

Heart rate variability (HRV) is a commonly used indicator for evaluating autonomic nerve function, and its high-frequency (HF) component mainly reflects vagal tone [51]. Multiple studies have found that obese patients have significantly reduced HF-HRV, indicating decreased vagal tone. Abnormal vagus nerve function in obese patients is closely related to binge eating behavior: the lower the vagal tone, the higher the frequency and severity of binge eating [52].

In addition, functional magnetic resonance imaging (fMRI) studies have found that, after eating, the activation degree of brain regions such as the NTS and hypothalamus in obese patients is significantly lower than that in normal-weight individuals, indicating that the central nervous system of obese patients has a weakened response to vagal afferent satiety signals [53]. These results indicate that abnormal vagus nerve function plays an important role in the occurrence and development of human obesity.

### 5.3. Bidirectional Vagal Circuitry and Microbiotic Modulation of NTS Signaling

Beyond the well-described afferent gut-to-brain satiety pathways, the vagus nerve forms a closed feedback loop via the dorsal vagal complex: NTS neurons integrate peripheral visceral signals and modulate parasympathetic output from the dorsal motor nucleus of the vagus (DMV). Vagal efferent fibers regulate gastrointestinal motility, secretory function, and enteroendocrine release of satiety hormones including GLP-1 and CCK, which in turn shape afferent signal input back to the NTS [37]. In parallel, gut microbiota and their bioactive metabolites (e.g., short-chain fatty acids, secondary bile acids) modulate vagal-NTS pathway activity: microbial products stimulate enteroendocrine hormone secretion and may directly alter vagal afferent excitability, whereas obesity-associated gut dysbiosis has been linked to impaired vagal signal transduction and blunted satiety responses [54,55]. These peripheral modulatory layers add mechanistic context to NTS-centered feeding regulation and may influence the therapeutic efficacy of NTS-targeted neuromodulation such as taVNS.

## 6. The Nucleus of the Solitary Tract (NTS): A Key Regulator of Feeding Homeostasis and Obesity

### 6.1. Physiological Functions of the Nucleus of the Solitary Tract

Located in the dorsal medulla oblongata, the NTS is the first entry point for visceral sensory information into the central nervous system and an important integration center for visceral-somatic and visceral-autonomic reflexes. In feeding regulation, the NTS is not only a passive relay station for peripheral vagal afferent signals, but also an active information processing center [1]. It can integrate various visceral signals from the gastrointestinal tract, cardiovascular system, and respiratory system, and receive descending regulatory signals from the hypothalamus, limbic system, and cerebral cortex. The integrated information is then projected to downstream autonomic nerve centers, neuroendocrine systems, and higher brain regions, thereby coordinating feeding behavior, gastrointestinal function, and energy metabolism [56].

Anatomically, the NTS can be divided into three subregions along the rostrocaudal axis of the medulla oblongata: rostral, intermediate, and caudal. Different subregions receive visceral afferents from different sources and have different functions [57]. The rostral and intermediate NTS mainly receive vagal afferent signals from the gastrointestinal tract and are the main subregions for feeding regulation; while the caudal NTS mainly receives afferent signals from the cardiovascular and respiratory systems. In terms of cell types, the NTS contains neurons with various neurotransmitter phenotypes, among which catecholaminergic neurons (expressing tyrosine hydroxylase, TH) are the most thoroughly studied. Recent studies have found that there is significant functional heterogeneity in NTS catecholaminergic neurons [1]: TH^+^/NPY co-expressing neurons located in the rostral and intermediate NTS can strongly promote feeding after activation; while the classic A2 noradrenergic neurons in the caudal NTS mainly play a role in inhibiting feeding.

In terms of hierarchical positioning, the NTS is located upstream of the hypothalamic feeding regulatory circuit. Peripheral vagal afferent signals first reach the NTS and, after preliminary integration, are transmitted to hypothalamic nuclei such as the ARC, PVH, and lateral hypothalamus (LH) through direct or indirect neural projections [58], thereby initiating or terminating feeding behavior. For example, NTS NPY/E neurons can project to the hypothalamic ARC, activating AgRP/NPY neurons to promote feeding; while NTS norepinephrine (NE) neurons can project to the PVH, activating PVH neurons to inhibit feeding [1]. In addition, the NTS can also regulate the rewarding effect and motivational behavior of food by projecting to the midbrain ventral tegmental area (VTA) and limbic system [56]. This hierarchical structure enables the NTS to act as a gateway for peripheral signals to enter the central feeding network, providing rapid and precise regulation of feeding behavior.

### 6.2. Functional Heterogeneity of NTS Neuron Subpopulations in Hunger and Satiety Regulation

The NTS is a highly heterogeneous nucleus containing multiple neuron subpopulations with different phenotypes. These neuron subpopulations have significant differences in anatomical distribution, neurotransmitter expression, receptor distribution, and function, and play different roles in hunger and satiety regulation respectively [59]. GLP-1ergic neurons are molecularly defined by the expression of glucagon-like peptide-1 (GLP-1), a bioactive peptide generated via tissue-specific post-translational cleavage of proglucagon encoded by the Gcg gene. Single-cell transcriptomic and electrophysiological validation confirms that the majority of these neurons co-express vesicular glutamate transporter 2 (VGlut2), classifying them as excitatory glutamatergic projection neurons. In female rats, photostimulation attenuates sucrose reward motivation, indicating dual regulation of homeostatic and hedonic feeding [60]. CCKergic neurons are marked by endogenous cholecystokinin (CCK) peptide expression, labeled via CCK-IRES-Cre mouse lines and concentrated in the dorsal and medial NTS subnuclei. Second, CCK type 1 receptor (CCK-1R)-positive neurons are defined by CCK-1R expression as direct cellular targets of peripheral CCK, with a broader NTS distribution and partial anatomical overlap with CCKergic neurons. Selective optogenetic or chemogenetic activation of NTS CCKergic neurons rapidly suppresses feeding and reduces meal size while inducing conditioned taste aversion (CTA), consistent with an aversive satiety pathway; targeted silencing of this population conversely increases meal size [61,62]. Catecholaminergic neurons are identified by expression of tyrosine hydroxylase (TH), the rate-limiting enzyme of catecholamine biosynthesis, corresponding to the well-characterized A2 noradrenergic and C2 adrenergic cell groups in the NTS [63].

#### 6.2.1. GLP-1ergic Neurons

GLP-1ergic neurons are mainly distributed in the caudal subnucleus (cNTS) of the NTS, especially the commissural, medial, and ventral subnuclei [64,65]. These neurons are glutamatergic, capable of synthesizing and releasing GLP-1, and also express other neurotransmitters such as CCK and TH.

GLP-1ergic neurons are a key neuron subpopulation in the regulation of feeding homeostasis. They can be activated by a variety of satiety signals [66], including gastric distension, CCK, leptin, and insulin. Activation of GLP-1ergic neurons can significantly inhibit feeding behavior, reduce food intake, and increase energy expenditure [56]. Conversely, inhibition of GLP-1ergic neurons leads to increased feeding and weight gain [67,68].

#### 6.2.2. CCK-Expressing and CCK Receptor-Positive Neurons

CCK-expressing neurons are mainly distributed in the caudal subnucleus of the NTS and the area postrema (AP). They are also glutamatergic neurons, and some are co-expressed with GLP-1ergic neurons [69]. CCK is an important intestinal hormone and also a neurotransmitter in the central nervous system, playing a key role in feeding regulation.

CCK neurons in the NTS can be activated by feeding-related signals, including gastric distension, intestinal nutrients, and GLP-1 receptor agonists [70]. By activating CCK1 receptors in vagal afferent neurons (VANs), CCK can significantly inhibit feeding behavior, reduce food intake and body weight [40]. Conversely, inhibition of CCK neuron sensitivity leads to increased feeding.

CCK neurons exert their feeding inhibitory effects mainly through projections to the PVH. Studies have found that CCK neurons send specific projections to the PVH, forming synaptic connections with neurons expressing melanocortin 4 receptor (MC4R) in the PVH, releasing CCK to activate these neurons and thereby produce satiety [22]. Notably, activation of CCK neurons can not only inhibit feeding but also encode positive reward signals, which is different from the role of GLP-1 neurons.

#### 6.2.3. Catecholaminergic Neuron Subpopulations

Catecholaminergic (CA) neurons, marked by tyrosine hydroxylase (TH) gene expression, show specific responses under fasting conditions and have obvious bidirectional regulatory effects [1]. In the hungry state, some CA neurons (such as NPY/E neurons) are activated by gastrointestinal signals to promote feeding behavior, while another type of CA neurons expressing dopamine β-hydroxylase (DBH) (such as NE neurons) respond to satiety signals, and their activation can inhibit feeding and project to the lateral parabrachial nucleus [71]. This finding helps to understand some seemingly contradictory phenomena of vagus nerve stimulation (VNS) in obesity and appetite intervention [1].

#### 6.2.4. Other Functional Neuron Subpopulations

In addition to the three main neuron subpopulations mentioned above, there are various other neuron subpopulations in the NTS involved in feeding regulation:

Leptin receptor (LepRb)-positive neurons: There are a large number of LepRb-expressing neurons in the NTS, which can directly perceive circulating leptin levels to regulate feeding behavior [72]. Leptin can activate LepRb-positive neurons in the NTS, enhance satiety signals [73], and inhibit feeding. In addition, leptin can also enhance the sensitivity of NTS neurons to intestinal hormones such as CCK and GLP-1 [40].

Oxytocin receptor (OXTR)-positive neurons: The NTS also expresses oxytocin receptors, and oxytocin can act on these receptors to inhibit feeding behavior [74]. Oxytocin neurons in the hypothalamic PVH project to the NTS, releasing oxytocin to activate OXTR-positive neurons in the NTS, regulating gastrointestinal motility and secretion functions while enhancing satiety signals [75].

GABAergic neurons: There are a large number of GABAergic neurons in the NTS, which mainly play an inhibitory regulatory role [37]. Recent studies have found that GABAergic neurons in the NTS project to the ARC, inhibiting AgRP/NPY neurons in the ARC and thereby inhibiting feeding behavior [76]. This finding challenges the traditional view that GABAergic neurons mainly promote feeding, indicating that GABAergic neurons in the NTS also play an important inhibitory role in feeding regulation [77].

## 7. Transcutaneous Auricular Vagus Nerve Stimulation (taVNS): A Non-Invasive Strategy Targeting the Nucleus of the Solitary Tract

Accumulating evidence supports a multi-level neural circuit model through which transcutaneous auricular vagus nerve stimulation (taVNS) may exert anti-obesity effects. At the anatomical level, taVNS targets the auricular branch of the vagus nerve (ABVN), whose afferent fibers project to the medullary NTS via the jugular-nodose ganglion. This neural pathway has been clearly confirmed by viral tracing techniques in animal experiments [78]. At the cellular level, preclinical studies in rodent models suggest that taVNS may preferentially modulate specific NTS neuronal subpopulations—including upregulation of satiety-promoting GLP-1ergic neurons and attenuation of aberrant catecholaminergic (TH^+^) neuronal activity. Within the proposed mechanistic framework, taVNS is proposed to modulate the activity of specific satiety-promoting NTS neuron subpopulations, including proglucagon (GCG)/GLP-1-expressing neurons and CCK-responsive neurons. Preproglucagon (PPG)/GLP-1ergic neurons in the NTS region are more inclined to respond to nutritional signals rather than aversive stimuli, and activation of these neurons can effectively induce satiety without adverse reactions such as nausea. Meanwhile, taVNS is hypothesized to inhibit the aberrant hyperactivation of tyrosine hydroxylase-positive (TH^+^) catecholaminergic neurons in the NTS—a population that is overactive in obesity and implicated in dysregulated feeding behavior. However, direct evidence for such selective neuronal modulation in humans remains indirect and limited, primarily inferred from neuroimaging and physiological studies. The following sections present the current evidence base for this proposed model, with clear indication of the experimental approach and species for each mechanistic claim.

### 7.1. Anatomical Basis

Transcutaneous auricular vagus nerve stimulation (taVNS) is a non-invasive neuromodulation technique whose theoretical basis lies in the fact that the auricular branch of the vagus nerve (ABVN) is the only branch of the vagus nerve distributed on the body surface [79]. Anatomical studies have shown that the ABVN originates from the jugular ganglion, runs along the posterior wall of the external auditory canal and the medial side of the auricle, and mainly innervates the skin sensation of the cymba conchae, concha cavum, posterior wall of the external auditory canal, and lateral surface of the tympanic membrane [80,81]. Among them, the skin of the cymba conchae region is almost completely innervated by the ABVN (innervation rate reaches 100%), while the innervation rates of the concha cavum, antihelix, and tragus are 45%, 73%, and 45% respectively [82].

The afferent fibers of the ABVN have the same central projection pattern as other visceral afferent fibers of the vagus nerve trunk: after entering the medulla oblongata through the jugular ganglion, their afferent impulses directly terminate in the medial and commissural subnuclei of the NTS, and are integrated in the same nucleus with afferent fibers from visceral organs such as the gastrointestinal tract, heart, and lungs [83]. This direct anatomical connection enables specific activation of NTS neurons through electrical stimulation of the ABVN on the body surface, thereby regulating autonomic nerve function and energy metabolism homeostasis of the central nervous system [84]. Compared with invasive VNS, taVNS avoids the risks and complications of surgical implantation, and has the advantages of simple operation, high safety, and good patient compliance [85], providing a new direction for non-pharmacological treatment of obesity and metabolic diseases.

### 7.2. Neuroimaging Evidence

Functional neuroimaging studies have provided direct human evidence for the specific regulatory effect of taVNS on the NTS. Functional magnetic resonance imaging (fMRI) studies have shown that when healthy subjects receive taVNS stimulation on the left cymba conchae, significant blood oxygen level-dependent (BOLD) signal enhancement appears in the NTS and DMV in the dorsal medulla oblongata, while stimulation of non-ABVN innervated areas of the auricle (such as the earlobe) does not observe similar brainstem activation [86]. A simultaneous taVNS-fMRI study by Badran et al. further confirmed that the cymba conchae is the optimal stimulation site for taVNS to activate the NTS, and its activation intensity is significantly higher than that of other sites such as the tragus and earlobe [79].

In addition to brainstem nuclei, taVNS can also regulate the activity of multiple higher brain regions related to feeding and metabolic regulation. Studies have found that taVNS can activate the PVH and dorsomedial nucleus (DMH) of the hypothalamus, which are key hubs for central energy metabolism regulation [87]; meanwhile, taVNS can also regulate the functional connectivity of the amygdala, insula, and prefrontal cortex, which are involved in the reward and emotional regulation of appetite. A study by Fang et al. showed that taVNS treatment in patients with depression can significantly change the functional connectivity between the NTS and the default network of the limbic system [88], suggesting that taVNS exerts extensive regulatory effects on the whole-brain network through the NTS-mediated ascending pathway.

While fMRI studies provide valuable in vivo evidence for taVNS modulation of brainstem activity, several methodological limitations should be considered when interpreting these findings. First, the NTS is a small nucleus (approximately 2–3 mm in diameter in humans), and standard fMRI spatial resolution (typically 2–3 mm isotropic) may not adequately resolve its fine structure, leading to partial volume effects with adjacent nuclei. Second, brainstem fMRI is particularly susceptible to physiological noise from cardiac and respiratory pulsations, which can obscure or mimic task-related BOLD signal changes.

### 7.3. Preclinical Evidence for Mechanistic Effects

The following section summarizes mechanistic evidence for taVNS-mediated anti-obesity effects, with a clear distinction between preclinical (animal) findings and clinical (human) observations. It is critical to note that most mechanistic insights into the proposed mechanism derive from rodent studies, where invasive recording and manipulation techniques enable precise characterization of neuronal circuits. Direct translation to humans requires careful consideration of anatomical differences (e.g., larger human brainstem, different auricular innervation patterns), physiological variability, and the technical limitations of non-invasive stimulation. Clinical evidence, while promising, remains preliminary and should be interpreted as hypothesis-generating rather than confirmatory.

Although preclinical studies of taVNS in the treatment of obesity are relatively few, existing evidence has initially confirmed its effectiveness in regulating feeding and metabolism. In a diet-induced obesity (DIO) rat model, 6 weeks of auricular vagus nerve stimulation (AVNS) treatment significantly reduced bilateral perirenal and epididymal white adipose tissue (WAT) mass and body weight in rats, while improving serum biochemical indicators [89]. The underlying mechanism is proposed to involve taVNS-mediated activation of GLP-1R-positive neurons in the NTS, which may upregulate hypothalamic POMC expression while concurrently reducing NPY and AgRP expression [27].

In addition, taVNS can also affect energy metabolism by regulating gastrointestinal function. Studies have found that VNS can delay gastric emptying, slow down small intestinal peristalsis, prolong the residence time of nutrients in the gastrointestinal tract, and thereby increase satiety [89]. In a high-fat diet-induced obese rat model, electrical vagus nerve stimulation reduced food intake and weight gain [90]. Some studies have proposed that low-frequency electrical stimulation of the gastric vagal branch may mimic endogenous vagal afferent satiety signals to induce satiety and modulate body weight; it is inferred that taVNS may exert analogous effects via a shared central pathway [91]. Existing studies have shown that taVNS can significantly affect the neural activity of hypothalamic feeding-related nuclei, including inhibiting the lateral hypothalamic nucleus and activating the ventromedial hypothalamic nucleus, to promote the formation and maintenance of satiety and reduce feeding [92,93].

### 7.4. Clinical Evidence

#### 7.4.1. Existing Supporting Evidence for Clinical Studies

At present, clinical studies of taVNS in obese populations are still in the preliminary stage, mainly small-sample, short-term studies. Accumulating clinical evidence indicates that taVNS may modulate gastrointestinal motility and increase the amplitude of gastric and duodenal peristaltic waves via activation of the dorsal vagal complex (including the NTS), which may in turn enhance satiety signaling [94]. In the fullness phase, food liking was significantly lower in the active group than in the sham group (stimulation × phase interaction *p* = 0.004; post hoc test *p* = 0.03). The reduction in liking from baseline to fullness was significantly larger in the active group (*p* = 0.03). In the randomized trial by Salaris & Azevedo (2025) [94], a significant stimulation × phase interaction effect was observed for food liking ratings (F(2, 4107) = 5.32, *p* = 0.004, partial η^2^ = 0.003); post hoc analysis confirmed that food liking was significantly lower in the active group than in the sham group during the fullness phase (z = −2.16, *p* = 0.03, Cohen’s d = 0.51, medium effect size). The magnitude of reduction in liking from baseline to fullness was significantly larger in the active group (mean difference = −0.14, 95% CI [−0.27, −0.01], t(68) = −2.13, *p* = 0.03, Cohen’s d = −0.51). In a small-sample randomized trial including 24 overweight or obese patients, 4 weeks of taVNS treatment produced significantly greater improvements in obesity indicators including body weight, BMI, and waist circumference compared with lifestyle intervention alone [95]. For body weight, the taVNS group showed a mean reduction of 2.57 ± 1.75 kg, compared with 0.24 ± 2.18 kg in the control group; the between-group difference was 2.33 kg (95% CI [0.63, 4.03]), with a large effect size (t = 2.86, *p* = 0.009, Cohen’s d = 1.17). For BMI, the between-group difference in change from baseline was 0.89 kg/m^2^ (95% CI [0.27, 1.51]), with a large effect size (t = 2.95, *p* = 0.007, Cohen’s d = 1.21). For waist circumference, the taVNS group showed a median reduction of 3.00 cm (IQR 3.00, 5.50), compared with 0.00 cm (IQR −1.00, 1.00) in the control group; the between-group difference was statistically significant with a large effect size (Z = 3.85, *p* < 0.001, r = 0.79). taVNS can reduce individuals’ expected preference for food and eating tendency in the satiety state; while in the hungry state, it reduces heart rate variability related to eating and food craving [96]. In the fasted state, cymba conchae stimulation significantly reduced rMSSD compared with sham (*p* = 0.036). Postprandially, rMSSD showed a physiological decrease in sham, tragus, and combined conditions, while no further decrease was observed in the cymba conchae condition, with a significant site × time interaction (*p* = 0.002). There was a main effect of time on mean gastric amplitude (MGA, *p* = 0.031), with a trend of increase in the cymba conchae group at 20–35 min postprandially, which did not survive multiple comparison correction. This suggests that taVNS may affect nutrient perception and feeding reward behavior through projections from the NTS to the parabrachial nucleus, thalamus, and dopaminergic midbrain (such as the VTA).

Meanwhile, taVNS also more widely affects the activity of different brain regions by regulating various neurotransmitters such as norepinephrine, acetylcholine, dopamine, and serotonin, thereby improving attention, decision-making behavior, and reward processing ability, and indirectly affecting feeding [86]. Compared with sham, verum stimulation significantly activated downstream targets of vagal afferents, including the nucleus of the solitary tract (NTS), dorsal raphe nucleus (DRN), substantia nigra (SN) and subthalamic nucleus (STN). All activated clusters survived cluster-level FWE correction (*p* < 0.05). taVNS can also improve gastrointestinal symptoms in diabetic patients [97], such as abdominal distension and early satiety, and improve patients’ quality of life.

#### 7.4.2. Insufficient and Reflective Clinical Research Evidence

However, there is still a lack of large-sample, long-term, multicenter randomized controlled trials to confirm the long-term efficacy and safety of taVNS in obesity management. Existing studies have small sample sizes, short follow-up times, and large differences in stimulation parameters, making it difficult to directly compare the results between different studies [98,99]. In addition, stimulation intensity and duration also affect the efficacy of taVNS, and the maximum sensory threshold tolerated by subjects is usually used as the optimal stimulation intensity [100].

Accumulating preclinical evidence suggests that taVNS may modulate NTS neuronal activity through the auricular branch of the vagus nerve, with preliminary findings indicating potential upregulation of satiety-promoting neuronal subpopulations (e.g., GLP-1ergic neurons) and attenuation of aberrant catecholaminergic neuronal activity in obesity models. These neurobiological effects have been associated with reduced homeostatic and hedonic feeding, decreased energy intake, and improved metabolic parameters in animal studies and small-scale human trials. However, it is important to note that direct evidence of taVNS-induced selective neuronal modulation in humans remains limited, and larger, well-controlled clinical trials are needed to confirm the efficacy, optimal parameters, and long-term sustainability of taVNS-mediated anti-obesity effects.

With respect to gastric interoception and ingestive behavior, a randomized controlled trial conducted by Salaris and Azevedo (2025) [94] in healthy adults found no significant between-group differences in gastric interoceptive accuracy (active: 56.25 ± 10.8 vs. sham: 55.76 ± 14.6; t(62.65) = 0.16, *p* = 0.87, BF_10_ = 0.24, moderate evidence for the null hypothesis), total water intake (active: 830.26 ± 347.7 mL vs. sham: 690.34 ± 263.8 mL; *p* = 0.07, BF_10_ = 0.97, anecdotal evidence), or water consumption at the satiety threshold following acute taVNS administration. Only a mild reduction in food liking was observed exclusively during the gastric fullness phase, while no changes were detected in core behavioral markers of food intake [94]. At the level of food motivational processing, Müller et al. (2021) [101] validated that acute taVNS exerts no significant effect on either hedonic (liking) or motivational (wanting) ratings of food stimuli in healthy participants. These findings failed to replicate the feeding-inhibitory effects of taVNS reported in earlier preliminary investigations [101]. Most published studies about taVNS have reported positive results, although this may reflect publication bias; studies with negative results may not have been published.

#### 7.4.3. Structured Evidence Appraisal

To facilitate evaluation of evidentiary strength and limitations, the current clinical evidence base for taVNS in appetite and weight regulation can be stratified along four key dimensions:

Approximately half of published human studies employ randomized controlled trial designs, with the remainder consisting of uncontrolled pre-post studies or crossover trials. Randomized designs provide stronger causal inference regarding stimulation-specific effects; however, most randomized trials are statistically underpowered due to small sample sizes, limiting the robustness of conclusions.

The literature comprises a mix of acute single-session stimulation paradigms and chronic multi-week intervention protocols. Acute studies are useful for probing immediate physiological effects on gastric function and neural processing, but cannot support conclusions regarding long-term weight loss efficacy or sustained metabolic benefits.

The majority of mechanistic and psychophysical studies recruit healthy adult participants, and only a small number of trials enroll overweight or obese patient populations. Findings derived from healthy individuals cannot be directly generalized to clinical obesity populations, which are characterized by altered vagal tone, leptin resistance, and dysregulated homeostatic feeding circuits.

Studies use a mix of objective metabolic endpoints (body weight, BMI, waist circumference, gastric motility) and subjective self-report measures (hunger, food liking, food craving). Objective endpoints provide more robust evidence of clinical benefit; however, most short-term studies rely on subjective ratings or physiological surrogate markers rather than sustained weight loss outcomes.

Collectively, the strongest available evidence comes from small randomized trials demonstrating short-term improvements in body weight and waist circumference in populations with obesity. Nevertheless, the overall evidence base remains insufficient to support definitive conclusions about the long-term clinical efficacy of taVNS for obesity management.

#### 7.4.4. Cross-Study Comparison of Stimulation Parameters

As summarized in Table 1, the included studies exhibit substantial heterogeneity across all core dimensions of stimulation protocols, which represents a primary barrier to cross-study comparison, result replication, and clinical translation. A systematic dimensional comparison reveals the following key sources of variability:

The most fundamental divide lies between invasive subdiaphragmatic/cervical VNS and non-invasive transcutaneous auricular VNS. Invasive preparations (Gil et al., 2011; Dai et al., 2020) [89,90] deliver electrical current directly to the vagal nerve trunk, enabling precise and robust activation of vagal afferents projecting to the nucleus of the solitary tract (NTS). In contrast, transcutaneous auricular stimulation targets only the auricular branch of the vagus nerve, with far more variable and indirect engagement of brainstem nuclei.

Even among taVNS studies, target sites are inconsistent. Two studies placed electrodes at the cymba conchae, the region with the densest auricular vagal innervation (Salaris & Azevedo, 2025; Müller et al., 2021) [94,101], whereas the clinical trial by Fan et al. (2024) [95] adopted traditional Chinese auricular acupoints (Spleen point, Endocrine point) without anatomical validation against vagal nerve distribution. These differences in target anatomy directly affect the proportion of activated vagal afferent fibers and the specificity of NTS modulation, confounding direct comparison of efficacy.

Reported stimulation frequencies range from 10 Hz to 40 Hz across preclinical invasive studies, and from 20 Hz to 25 Hz across human taVNS studies. Notably, Fan et al. (2024) [95] employed a variable dense-disperse wave pattern rather than fixed-frequency continuous stimulation.

Frequency is a critical determinant of neural recruitment and circuit-level effects. Preclinical neurophysiology indicates that low-frequency stimulation preferentially activates unmyelinated C-fiber afferents that mediate visceral satiety signaling, while higher frequencies may engage myelinated A-fibers or exert mixed excitatory-inhibitory effects on NTS circuits. To date, no head-to-head study has systematically compared the anti-obesity efficacy of different frequencies in either animals or humans, and there is no consensus on an optimal frequency range for NTS-mediated appetite regulation.

Pulse width varies by over 50-fold across studies: from 200 µs in human taVNS protocols to 10 ms in the invasive rat study by Gil et al. (2011) [90]. Pulse width is a key determinant of fiber-type selectivity: shorter pulse widths (<1 ms) preferentially activate large myelinated fibers, while longer pulse widths are required to recruit small unmyelinated vagal afferent C-fibers—the primary conductors of gastrointestinal satiety signals to the NTS.

Intensity calibration methods are also highly inconsistent. Invasive animal studies use fixed current or voltage amplitudes (200 mV constant voltage in Gil et al., 2011 [90]; 6 mA constant current in Dai et al., 2020 [89]), whereas human taVNS studies employ a range of titration strategies: some set intensity below the perceptual threshold to avoid somatosensory confounds (Salaris & Azevedo, 2025) [94], others titrate to a just-perceptible tingling sensation (Müller et al., 2021) [101], and clinical protocols use subjectively tolerable intensity levels (Fan et al., 2024) [95]. This variability introduces substantial inter-individual and inter-study differences in the magnitude of neural activation, making it impossible to isolate reliable dose–response relationships from the existing literature.

Stimulation delivery patterns range from continuous output (Gil et al., 2011; Dai et al., 2020; Salaris & Azevedo, 2025) [89,90,94] to intermittent 30 s ON/30 s OFF cycling (Müller et al., 2021) [101], and to twice-daily 30 min sessions (Fan et al., 2024) [95]. Duty cycle determines total daily stimulation dose and may influence neuroplastic adaptation with chronic use.

Equally importantly, total treatment duration spans from single acute sessions to 6 weeks of chronic intervention. Acute stimulation studies can only probe immediate physiological or subjective effects on gastric function and food appraisal, while chronic protocols are required to assess sustained effects on body weight and metabolic outcomes. This mismatch in treatment duration explains much of the discrepancy in reported outcomes: chronic invasive VNS consistently reduces food intake and body weight in animals, whereas acute human taVNS studies yield mostly null or weak effects on subjective appetite ratings.

### 7.5. Critical Knowledge Gaps and Research Challenges

Despite preliminary evidence supporting the anti-obesity potential of taVNS, key knowledge gaps and methodological barriers remain that substantially hinder its clinical translation.

#### 7.5.1. Uncertainty in taVNS Stimulation Parameter Optimization

No consensus has been reached on standardized stimulation parameters for obesity-specific taVNS. Core variables including frequency, electrode location, current intensity, and treatment duration vary markedly across studies, impeding cross-study comparison and clinical generalization. Both low-frequency (1–10 Hz) and high-frequency (20–50 Hz) protocols have been applied in metabolic research, but no head-to-head trials have identified the optimal frequency profile for NTS satiety circuit modulation [98].

To date, only minimum reporting standards for taVNS research have been issued internationally, with no disease-specific parameter recommendations for obesity [98].

#### 7.5.2. Lack of Direct Evidence for Selective Modulation of NTS Neuronal Subpopulations

The mechanistic hypothesis that taVNS selectively modulates distinct NTS neuronal subtypes is predominantly derived from preclinical rodent studies, with no direct confirmatory evidence in humans. Invasive optogenetic and chemogenetic tools enable precise dissection of this mechanistic hypothesis in animal models, but such approaches are not feasible in human research. Current human in vivo evidence relies entirely on functional neuroimaging, which has inherent technical limitations in brainstem research [86].

#### 7.5.3. Long-Term Efficacy and Tolerance Issues

Available clinical evidence is limited to small-sample, short-term pilot studies. Systematic scoping reviews indicate that short-term taVNS intervention can significantly reduce body weight, BMI and waist circumference compared with sham control [99], but no follow-up data beyond 3 months are available, leaving the durability of weight loss uncharacterized. Experience with implanted VNS devices indicates that chronic electrical stimulation may induce neuroadaptive changes and gradual therapeutic tolerance, but whether non-invasive taVNS presents a similar tolerance profile with long-term daily use remains uninvestigated [91].

#### 7.5.4. Safety Profile

taVNS is generally well-tolerated as a non-invasive intervention, but long-term safety data in obese populations remain insufficient. Reported adverse events: The majority of adverse events are mild, transient local reactions at the electrode site, including tingling, pruritus and erythema. Systemic events are rare, including mild dizziness, headache and nausea, which typically resolve after intensity reduction. No serious treatment-related adverse events have been consistently reported across systematic reviews [99,102].

## 8. Conclusions and Prospects

In long-term high-fat-diet-induced obesity models, the sensitivity of the NTS to peripheral satiety hormone signals such as GLP-1 and CCK from the gastrointestinal tract is significantly reduced, leading to imbalance in the homeostatic feeding regulatory mechanism. The proposed mechanism of taVNS as an anti-obesity intervention is biologically plausible, supported by convergent anatomical, electrophysiological, and neuroimaging evidence demonstrating that vagal afferent signaling modulates NTS activity and influences both homeostatic and hedonic feeding pathways. Preclinical studies in animal models provide preliminary support for the hypothesis that taVNS may enhance NTS integration of satiety signals, which may in turn reduce energy intake and support weight reduction. However, it is critical to emphasize that a causal link between taVNS-induced NTS modulation and sustained weight loss in humans has not been definitively established. Current clinical evidence, while suggestive of beneficial effects on appetite regulation and metabolic parameters, does not conclusively demonstrate that these effects are mediated specifically through NTS pathways, nor do they confirm long-term efficacy for weight management. Further research employing targeted neuroscientific approaches (e.g., fMRI with brainstem-specific sequences, pharmacological challenge paradigms) and larger clinical trials with mechanistic endpoints are needed to establish the causal pathways and clinical significance of taVNS-mediated NTS modulation in obesity.

Future research testing this mechanistic hypothesis should focus on systematic optimization of taVNS stimulation parameters. Using neuroimaging techniques such as functional magnetic resonance imaging (fMRI) and electroencephalography (EEG) combined with optogenetic and chemogenetic methods in animal models to screen the optimal combination of stimulation parameters that can specifically activate satiety-promoting neuron subpopulations in the NTS. Establish a parameter optimization platform based on neuroimaging biomarkers to achieve “on-demand regulation” of precise stimulation. At the same time, the influence of individual differences should be fully considered, and personalized taVNS intervention programs should be developed according to patients’ age, gender, obesity subtype, genetic background, and neurophysiological characteristics. Systematic optimization of five core parameters (frequency, pulse width, intensity, location, duration) is the foundation of clinical translation. Multi-arm randomized dose-finding trials should be conducted, using neurophysiological and metabolic biomarkers as primary endpoints to characterize dose–response relationships and identify optimal parameter sets for obesity. Standardized parameter reporting guidelines should be universally adopted to enable cross-study data pooling and evidence synthesis [98]. Obesity is a biologically heterogeneous disorder, and uniform protocols cannot achieve consistent benefits across all patients. Stratification strategies should be developed along three dimensions: (1) phenotypic stratification: taVNS is preferentially indicated for patients with impaired vagal satiety signaling and gut–brain axis dysfunction; (2) comorbidity-based stratification: parameters should be adjusted for patients with type 2 diabetes, metabolic syndrome or binge-eating disorder; (3) biomarker-guided personalization: baseline vagal tone (heart rate variability), peripheral gut hormone profiles and brainstem neuroimaging biomarkers can serve as predictive indicators for treatment response to guide individualized parameter titration. In addition, the research and development of new taVNS devices, such as wearable and closed-loop stimulation systems, should be explored to improve the convenience and compliance of treatment.

## Figures and Tables

**Table 1:** Summary of Key Preclinical and Clinical Studies Investigating Vagus Nerve Stimulation for Feeding Regulation and Obesity.

Study ID	Study Type	Model/Population	Sample Size	Stimulation Site	Core Stimulation Parameters	Intervention Duration	Control/Sham Condition	Primary Outcomes	Key Limitations
**Preclinical studies**									
Gil et al., 2011 J Physiol Pharmacol [90]	In vivo parallel-group animal experiment	High-fat diet-induced obese male Wistar rats	Total *n* = 24, 3 groups (*n* = 8 per group): active VNS, sham surgery, intact control	Left subdiaphragmatic vagus nerve (invasive implant)	10 Hz, 10 ms pulse width, 200 mV constant voltage, rectangular pulses	42 days (6 weeks), 12 h/day (dark phase)	Sham group: implanted inactive microstimulator without electrode wrapping; intact control group with no surgery	1. Total food intake significantly reduced in VNS vs. sham (*p* = 0.042) 2. Body weight gain reduced (21.1% vs. 30.5% in sham) 3. Epididymal fat pad weight significantly decreased4. c-Fos positive neurons in NTS bilaterally increased5. Serum ghrelin elevated, leptin decreased; nesfatin-1 non-significantly elevated	Invasive cervical VNS (not taVNS); only male rats; small sample size; no blinding; no neuronal subtype-level mechanistic validation
Dai et al., 2020 Obesity Surgery [89]	In vivo parallel-group animal experiment	High-fat diet-induced obese (DIO) male Sprague–Dawley rats	DIO rats: *n* = 16, 2 groups (*n* = 8 per group): active VNS, sham-VNS; additional 10 normal control rats	Bilateral subdiaphragmatic vagal nerves (invasive implant)	40 Hz, continuous output, 0.2 ms pulse width, 6 mA constant current	4 weeks, 2 h/day (during feeding period), 5 days/week	Sham-VNS: electrodes implanted but no current delivered	1. Daily food intake reduced by ~33% vs. sham (*p* < 0.001) 2. Progressive body weight reduction over 4 weeks 3. Increased vagal activity (HF-HRV) and reduced sympathovagal ratio 4. Elevated plasma GLP-1, PYY, and pancreatic polypeptide 5. Delayed solid gastric emptying, increased gastric volume	Invasive VNS (not taVNS); only male rats; small sample size; no direct NTS neuronal recording; short observation period
**Clinical studies**									
Salaris & Azevedo, 2025 Psychophysiology [94]	Randomized, single-blind, between-subjects controlled trial	Healthy young adults (university students, normal BMI)	Analyzed *n* = 70; active taVNS *n* = 35, sham *n* = 35	Left cymba conchae	24 Hz, 200 μs pulse width, intensity set below individual perceptual threshold	Acute single session (continuous stimulation throughout tasks)	Sham stimulation at left earlobe	1. No significant difference in gastric interoceptive accuracy (*p* = 0.87) 2. No significant difference in total water intake 3. Significant reduction in food liking ratings during the fullness phase in active vs. sham (*p* = 0.03) 4. No significant effect on food wanting	Healthy population only; acute single stimulation; predominantly female sample; no long-term metabolic outcomes; earlobe sham specificity debated
Müller et al., 2021 bioRxiv (preprint) [101]	Randomized, single-blind, within-subjects crossover trial	Healthy adults, normal BMI range	Analyzed *n* = 82; left-side taVNS *n* = 42, right-side *n* = 40	Cymba conchae (separate left/right subgroups)	25 Hz, biphasic, 30 s ON/30 s OFF duty cycle, intensity titrated to tingling sensation below pain threshold	Acute single session	Sham: device placed upside-down on earlobe	1. No significant effect on food liking ratings (*p* = 0.513) 2. No significant effect on food wanting ratings (*p* = 0.113) 3. Bayesian analysis provided moderate evidence for the null hypothesis4. No subgroup effects by stimulation side, sex, or food type	Healthy population only; acute stimulation; preprint not formally peer-reviewed; only subjective cue-reactivity outcomes
Fan et al., 2024 J Guangzhou Univ Chin Med [95]	Parallel-group randomized controlled trial (add-on design)	Overweight/obese Chinese adults (BMI 24–32.5 kg/m^2^)	Analyzed *n* = 24; taVNS + lifestyle *n* = 11, lifestyle control *n* = 13	Unilateral ear acupoints (Spleen point, Endocrine point)	Dense-disperse wave, 20 Hz, 9 V, 30 min per session	4 weeks, twice daily (morning + evening), 5 days/week	Lifestyle-only control (calorie-restricted diet intervention); no sham-taVNS control	1. Significant reductions in body weight, BMI, and waist circumference in taVNS group vs. control (*p* < 0.01 for BMI difference) 2. Increased ReHo in left prefrontal lobe/medial frontal gyrus 3. Decreased ReHo in right parietal lobe	No sham stimulation control; small sample size; single-center; non-standard auricular acupoint site; no participant/assessor blinding

Note. Studies exhibit substantial methodological heterogeneity in design, population, stimulation parameters, and outcome measures. This table provides a descriptive summary only and does not support quantitative cross-study comparison of efficacy.

## Data Availability

No new data were created or analyzed in this study. Data sharing is not applicable to this article.
